# Injury of the Vestibulocerebellar Tract and Signs of Ataxia in Patients with Cerebellar Stroke

**DOI:** 10.3390/jcm12216877

**Published:** 2023-10-31

**Authors:** Sang-Seok Yeo, Seung-Min Nam, In-Hee Cho

**Affiliations:** 1 Department of Physical Therapy, College of Health Sciences, Dankook University, 119, Dandae-ro, Dongnam-gu, Cheonan-si 31116, Chungnam, Republic of Korea; eangbul@hanmail.net; 2 Department of Sports Rehabilitation and Exercise Management, Yeungnam University College, 170, Hyeonchung-ro, Nam-gu, Daegu 42415, Gyeongsangbuk-do, Republic of Korea; ngd1339@naver.com; 3 Department of Health, Graduate School, Dankook University, 119, Dandae-ro, Dongnam-gu, Cheonan-si 31116, Chungnam, Republic of Korea

**Keywords:** cerebellum injury, vestibulocerebellar tract, ataxia, Purdue pegboard test, diffusion tensor imaging

## Abstract

Background: The vestibulocerebellar tract (VCT) is responsible for maintaining balance, spatial orientation, and coordination. Damage to the vestibular system is accompanied by symptoms of balance disorder or ataxia. This study aimed to compare cerebellar dysfunction according to VCT damage in patients with cerebellar stroke. Methods: Six patients with cerebellum injury were recruited. This study measured ataxia and hand function related to visuomotor integration and manual dexterity using the Purdue pegboard test. The primary and bilateral secondary VCTs were reconstructed to investigate the integrity of pathways using diffusion tensor imaging (DTI). Results: The ataxia sign was positive in five patients (83%) at onset. In the result of the pegboard test, all patients had hand dysfunction in the dominant hand (100%). Likewise, all patients also had non-dominant hand dysfunction (100%). On the DTI tractography, the left and right primary VCTs of the patients demonstrated a 25% injury rate. Furthermore, the injury rates of ipsilateral and contralateral secondary VCTs were 50% and 58%. Conclusions: Ataxia is related to secondary VCTs, and hand dysfunction is also related to VCTs. Therefore, we believe that the current study will be helpful in evaluating and providing a clinical intervention strategy for patients with ataxia and hand dysfunction following cerebellar injury.

## 1. Introduction

The cerebellum is located inferior to the cerebrum and dorsal brainstem, and consists of two hemispheres within the cerebellar cortex [1]. The cerebellum is responsible for the motor coordination of limb movement, balance, posture control, and motor learning [2,3]. In addition, the cerebellum also plays a role in various functions that are not related the movement such as working memory, motion perception, and spatial processing [4]. Cerebellar stroke is a relatively rare brain injury that accounts for approximately 10% of all strokes [5]. Patients with cerebellar stroke commonly experience loss of coordination, balance problems, nausea, and vomiting, which can be caused by vestibular dysfunction following cerebellar damage [6,7]. Additionally, due to cerebellar dysfunction, symptoms of cerebellar ataxia may appear, in which balance, gait, and limb and eye movements cannot be coordinated [8,9].

Ataxia is a problem with voluntary movement control caused by abnormalities in muscle and motor coordination functions [8,9,10]. The symptoms of ataxia include problems with precise movement control while reaching, manipulating objects, or writing [11]. Additionally, balance problems in the standing position and gait disturbances can be observed in patients with truncal ataxia [12]. Ataxia is caused by damage to the parts of the brain related to coordination, and is often observed in patients with cerebellar injuries [12]. However, patients with brainstem lesions such as pontine hemorrhage can show ataxia and coordination problems without injury to the cerebellar region [13,14]. These symptoms may be caused by damage to the connectivity between the cerebellum and pons, which is related to coordination [13,14]. 

The pegboard test is a widely used neurological assessment tool that measures fine motor dexterity and hand–eye coordination. Many studies have investigated the relation between neural tract and hand function using the pegboard test. As the results show, it has been demonstrated that the corticospinal tract (CST) regulates hand function, and many studies are still being conducted to investigate the changes of hand function according to the degree of CST reconstruction using brain imaging techniques [15,16,17]. However, several recent studies reported that hand function was affected by spatial orientation and motor coordination, which were functions controlled by the cerebellum [4,18,19,20]. In their studies, Küper et al. investigated the reaching and grasping functions in patients following cerebellar stroke because the cerebellar injury made it difficult to perform motor functions that required fast movement and spatial coordination [19,20]. As the results show, the impairment of hand function including reaching and grasping in patients following cerebellar stroke was related to the superior and inferior cerebellar cortex [19,20]. In addition, Barbuto et al. demonstrated the association of hand function using simple reaching tasks and the pegboard test with adults that showed severe ataxia following degenerative cerebellar diseases. They reported that hand function using these tasks and test were correlated with ataxia severity [18]. However, there is a lack of research comparing hand function using the Purdue pegboard test with the specific regions and neural pathways of cerebellum. The vestibulocerebellar tract (VCT) is a neural pathway that connects the vestibular nucleus to the uvula nodulus of the cerebellum, and is responsible for maintaining balance, spatial orientation, and coordination [21]. In general, the vestibular system detects changes in head position and movement, and controls static and dynamic balance based on vestibular sensory information [21,22]. Damage to the vestibular system is accompanied by an impairment of the sense of position and movement, and symptoms of balance disorder or ataxia [23,24,25]. Recently, a study on the signs of ataxia following VCT injury in patients with cerebellar hemorrhage was reported [26]. In addition, a previous study reported on severe ataxia in a patient with pontine hemorrhage due to an injury of the spinocerebellar tract and cerebellar peduncle, etc. [14]. Therefore, assessing neural connectivity in patients with damage to the cerebellum or brainstem region is important for the initial evaluation and prognosis of ataxia [27].

Diffusion tensor imaging (DTI) tractography enables anatomical structures and functional connectivity to be visualized and reconstructed by imaging water diffusion patterns [28,29,30]. DTI tractography provides images of the diffusion properties of white matter by quantifying diffusion in multiple directions [28,29,30]. In particular, the primary and bilateral secondary VCTs have been studied in normal healthy adults and patients with brain hemorrhage for investigating the anatomical structures of VCTs using DTI tractography. However, few studies have investigated VCTs and progressed the reconstruction of VCTs in patients with cerebral and cerebellar stroke. In addition, no study has investigated the relation between ataxia, hand function, and the degree of VCT reconstruction. 

Therefore, in the current study, we aimed to compare the differences in cerebellar dysfunction symptoms such as ataxia and hand function using the Purdue pegboard test according to VCT damage in patients with cerebellar stroke. 

## 2. Material and Methods

### 2.1. Subjects

Six patients presenting with quadriparesis due to cerebellum injury on magnetic resonance imaging (MRI) were recruited for this study. The inclusion criteria were as follows: (1) first-ever cerebellar injury; (2) no past diagnosis associated with vestibular function; and (3) no history of musculoskeletal, neurological, or cognitive dysfunction. All participants provided informed consent before undergoing DTI tractography and functional evaluations. This study was approved by the institutional review board of Dankook University.

### 2.2. Clinical Evaluation

This study checked that there were ataxia symptoms in each patient, and evaluated ataxia and hand function using the Purdue pegboard test after an infarction or intracerebral hemorrhage (ICH) in the cerebellum. The Purdue pegboard test measures hand function related to visuomotor integration and manual dexterity, and requires motor coordination and spatial orientation [31,32,33]. The participants placed pegs from a cup into a board on which 25 small holes were arranged, and recorded the number of pegs they were able to place as fast as possible within 30 s. This was performed separately using the dominant and non-dominant hands. The procedure was conducted three times and the average score of pins was calculated. The normal score range of healthy men aged 30–85 was from 11 to 16 with their dominant hand, and from 10 to 15 with their non-dominant hand. For women, the normal range for the same age was from 13 to 16 with their dominant hand, and from 11 to 15 with their non-dominant hand.

### 2.3. Diffusion Tensor Image Tractography

The data of the DTI tractography were acquired using a 6-channel head coil on a 1.5 T Philips Gyroscan Intera (Philips, Best, The Netherlands) with single-shot echoplanar imaging. For each of the 32 non-collinear diffusion-sensitizing gradients, 67 contiguous slices parallel to the anterior commissure-posterior commissure line were collected. The imaging parameters were as follows: acquisition matrix = 96 × 96; reconstructed matrix = 192 × 192; field of view = 240 × 240 mm^2^; TR = 10,726 ms; TE = 76 ms; parallel imaging reduction factor (SENSE factor) = 2; EPI factor = 49; b = 1000 s/mm^2^; NEX = 1; and a slice thickness of 2.5 mm with no gap (acquired voxel size 1.3 × 1.3 × 2.5 mm^3^) [34].

### 2.4. Probabilistic Fiber Tracking

Diffusion-weighted imaging data were analyzed using the Oxford Centre for Functional Magnetic Resonance Imaging of the Brain (FMRIB) Software Library (FSL; https://www.fmrib.ox.ac.uk/fsl, Analysis Group, Oxford, UK, accessed on 25 April 2023). The head-motion effect and image distortions owing to eddy currents were corrected using affine multiscale two-dimensional registration. Fiber tracking was performed using a probabilistic image method based on a multifiber model and utilizing image routines implemented in FMRIB diffusion (5000 streamline samples, 0.5 mm step length, curvature threshold = 0.2) [35]. The primary and secondary VCTs and CST were determined by selecting fibers passing through a single-seed region of interest (ROI) and the target ROI. The seed and target ROI of the VCTs were located as follows: primary VCT, seed ROI: the superior and medial vestibular nuclei at the pons level [36]; and secondary VCTs, seed ROI: the inferior and medial vestibular nuclei at the medulla oblongata level. The target ROIs of the primary and secondary VCTs were, respectively, placed on the ipsilateral and bilateral uvula-noduli of the cerebellum [36,37]. To reconstruct the CST, the seed ROI was placed on the CST portion of the pontomedullary junction on a color map, and the target ROIs were placed on the CST portion of the anterior mid-pons and the primary motor cortex [38]. A total of 5000 samples were generated from a seed voxel, and the results were visualized at a minimum of one streamline for each voxel. This study confirmed whether the CST and primary and bilateral secondary VCTs were reconstructed, and calculated the injury rate according to the results of reconstruction using DTI tractography.

## 3. Results

The characteristics of the six patients (five males, one female; mean age 61.83 ± 18.76 years) are shown in Table 1. Two patients had spontaneous ICH (S-ICH) in the bilateral cerebellum, and one had S-ICH in the right cerebellum. Three patients had cerebellar infarctions in the left and right hemispheres. 

### 3.1. Clinical Evaluation

The results of symptoms according to clinical evaluation following cerebellar stroke are shown in Table 1. The ataxia sign was positive in five patients (5/6, 83%) and negative in one patient at onset. The dominant hand in all patients was the right hand, and hand function was examined using the Purdue pegboard test. As the results show, the mean of the pegboard score in the dominant hand was 6.17 ± 2.79, and all patients had hand dysfunction (100%) (Table 1). Likewise, all patients also had non-dominant hand dysfunction (100%), with a mean pegboard score of 5.33 ± 3.08. Additionally, dysarthria signs appeared in two patients (2/6, 33%), and nystagmus, diplopia, and dysphagia signs were negative in all patients (Table 1).

### 3.2. Diffusion Tensor Imaging

**Figure 1:**
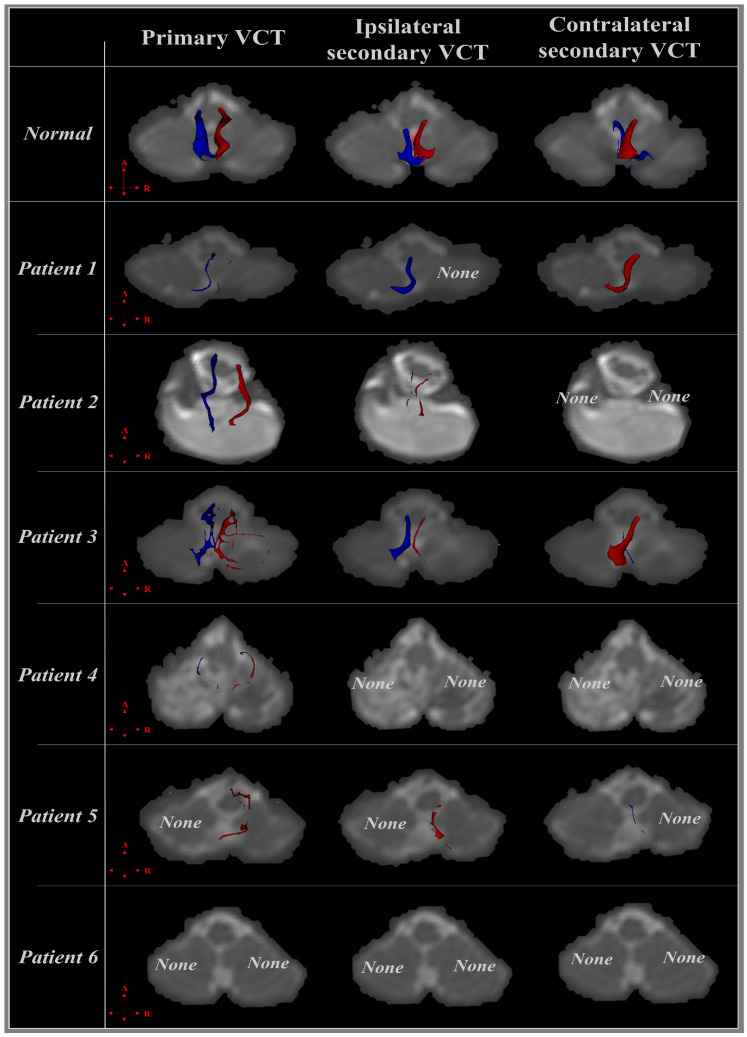
The vestibulocelebellar tract (VCT) is shown in a normal healthy adult and six patients according to diffusion tensor imaging (DTI) tractography. The right (red) and left VCTs (blue) are shown in the transverse plane, and the integrity of six patients suffering from spontaneous-intracerebral hemorrhage or infarction of the cerebellum is shown using DTI tractography. Among the primary and bilateral VCTs of each patient, the tract that was not completely reconstructed was marked as “none”.

The injury rate of primary and secondary VCTs and CST in the patients with cerebellar injury are shown in Table 2. In addition, Figure 1 show the integrity and projected direction of primary and bilateral secondary VCTs using DTI tractography. For the DTI tractography, the CSTs of all patients were reconstructed (100%). In the case of VCTs, the primary VCT was reconstructed in 8 out of 12 cases of the left and right primary VCTs in the patients, and calculated at a 25% injury rate (3/12). Furthermore, the ipsilateral secondary VCT was reconstructed in 6 out of 12 cases of left and right ipsilateral secondary VCTs, and calculated at a 50% injury rate (6/12), and the contralateral VCT was reconstructed in 5 out of 12 cases of left and right contralateral secondary VCTs, and showed at a 58% injury rate (7/12) (Table 2, Figure 1).

#### 3.2.1. Patient 1

An 82-year-old, right-handed male was diagnosed with spontaneous-intracerebral hemorrhage (S-ICH) in the right cerebellar hemisphere on a T2-weighted brain MRI. The patient showed ataxia and both hand function impairment despite the absence of damage to the corticospinal tract (CST) on the DTI tractography (Table 2). Although the left primary and ipsilateral secondary VCTs were less injured, the integrity of the left contralateral secondary VCT was not preserved because the contralateral secondary VCT was projected to the opposite side of the cerebellar hemisphere. In contrast, the integrity of the right primary VCT was not preserved, and the ipsilateral secondary VCT appeared to be completely injured due to a right cerebellar hemorrhage according to the result of the DTI tractography (Table 2, Figure 1).

#### 3.2.2. Patient 2

A 31-year-old, right-handed male. The T2-weighted brain MRI showed ICH in the cerebellum. The patient showed ataxia, dizziness, and both hand function impairment despite the absence of damage to the CST on the DTI tractography (Table 1 and Table 2). In the case of the left VCTs, the primary VCT was projected from the vestibular nuclei at the pons level to uvula-nodulus, and the integrity was preserved. In contrast, the tract volume of the ipsilateral secondary VCT was very narrowing, and the integrity was not maintained. Although the right primary and ipsilateral secondary VCTs showed a difference of tract volume compared to the left VCTs, the reconstruction pattern of the VCTs was similar. However, the left and right contralateral secondary VCTs were completely injured because neither contralateral VCT was observed on the DTI tractography (Table 2, Figure 1). 

#### 3.2.3. Patient 3

A 64-year-old, right-handed female was diagnosed with S-ICH in the cerebellum on a T2-weighted brain MRI. The patient showed ataxia, and although the CST was not affected by cerebellar hemorrhage, she complained of a dysfunction of both hands (Table 1). Although there were differences in the degree of integrity, the primary and bilateral secondary VCTs were reconstructed in the results of the VCTs (Table 2). The integrity of the left and right primary VCTs was not preserved compared to the ipsilateral secondary VCTs. In contrast, the ipsilateral and contralateral secondary VCTs were preserved, but the tract volume of the secondary VCTs projected to the right uvula-nodulus was lower compared to the secondary VCTs projected opposite the cerebellar region (Figure 1).

#### 3.2.4. Patient 4

A 67-year-old, right-handed male. The T2-weighted brain MRI showed an infarct in both cerebellar hemispheres. Although the patient did not show the ataxia symptom, he complained of hand function impairment despite no damage to the CST (Table 1 and Table 2). According to the DTI tractography results, the integrity of both primary VCTs was low, and the secondary VCTs were completely damaged because neither left nor right secondary VCTs were observed on the DTI tractography (Table 2, Figure 1). 

#### 3.2.5. Patient 5

A 77-year-old, right-handed male was diagnosed with an infarct in the bilateral cerebellar hemispheres on a T2-weighted brain MRI. The patient complained of ataxia, and although the CST was not injured on the DTI tractography, he showed mild hand dysfunction, particularly in the left hand rather than the right hand (Table 1). In the right VCTs, although the integrity of the right primary and ipsilateral secondary VCTs was decreased, they were not severely affected because the VCTs were not narrowing on the DTI tractography. However, the contralateral secondary VCT was not observed on the DTI tractography. Furthermore, the left primary and ipsilateral secondary VCTs also appeared to be completely injured. The left contralateral secondary VCT could be observed, but the integrity was not preserved, and the tract volume was narrowing (Table 2, Figure 1). 

#### 3.2.6. Patient 6

A 50-year-old, right-handed male. A T2-weighted brain MRI showed an infarct in both cerebellar hemispheres. He showed the ataxia symptom, and complained of function impairment of both hands despite no damage to the CST (Table 1, Table 2). According to the DTI tractography results, all VCTs were completely damaged because the left and right secondary VCTs were not reconstructed on the DTI tractography (Table 2, Figure 1).

**Table 1:** Demographics of patients with quadriparesis following cerebellar injury.

Patient	Sex/Age (Year)	Onset Duration	Diagnosis	Lesion on MRI	Cerebellar Symptoms					Purdue Pegboard (Score)	
					Ataxia	Nystagmus	Dysarthria	Dysphagia	Diplopia	Dominant	Non-Dominant
1	M/82	29 days	S-ICH	Rt.	+	−	−	−	−	5	6
2	M/31	65 days	S-ICH	both	+	−	+	−	−	8	7
3	F/64	12 days	S-ICH	both	+	−	−	−	−	7	8
4	M/67	44 days	Infarction	both	−	−	+	−	−	2	1
5	M/77	17 days	Infarction	both	+	−	−	−	−	10	8
6	M/50	16 days	Infarction	both	+	−	−	−	−	5	2

+, positive sign; −, negative sign; M, male; F, female; MRI, magnetic resonance imaging; S-ICH, spontaneous-intracerebral hemorrhage.

**Table 2:** Results of DTI integrity of the CST, and primary and secondary VCTs in patients with cerebellar injury.

Patient		CST	Primary VCT	Ipsilateral Secondary VCT	Contralateral Secondary VCT
1	R	+	+	−	+
	L	+	+	+	+
2	R	+	+	−	−
	L	+	+	−	−
3	R	+	+	+	−
	L	+	−	−	+
4	R	+	+	+	−
	L	+	+	+	−
5	R	+	+	+	+
	L	+	+	+	+
6	R	+	−	−	−
	L	+	−	−	−
Injury rate (%)		0.00	25.00	50.00	58.33

DTI, diffusion tensor imaging, CST, corticospinal tract; VCT, vestibulocerebellar tract; R, right; L, left.

## 4. Discussion

In this study, we investigated cerebellar symptoms, such as ataxia, and Purdue pegboard test results related to the fine motor function of the hands in six patients with cerebellar stroke. In addition, analyses of the CST and primary and secondary VCTs were performed using DTI tractography to confirm the pattern of neurological injury due to cerebellar stroke. All patients showed intact CST integrity in both hemispheres. In contrast, the damage rate of the primary VCT was 25%, while those of the ipsilateral and contralateral secondary VCT were 50% and 58%, respectively. Regarding cerebellar symptoms, 83% of patients showed ataxia and 33% showed dysarthria; however, signs of nystagmus, dysphagia, and diplopia were not observed in any patient. The results of the Purdue pegboard test showed a 100% decrease compared to the performance scores of healthy adults presented in a previous study [39].

Cerebellar ataxia is induced by cerebellar injury, and affects motor coordination, balance, and movement control [40,41]. Although balance and gait are controlled by various factors, such as the pathways of the spinocerebellum, vestibulocerebellum, and cerebrocerellum, the vestibulocerebellar tract of the vestibulocerebellum is commonly regarded as an important factor [26,42]. Barmack (2003) reviewed the central vestibular system, which is related to the vestibular nuclei and the cerebellum. Secondary vestibular mossy fibers are affected by damage to the uvula-nodulus of the cerebellum, which influences postural and optokinetic reflexes [37]. Therefore, the secondary VCT carries information related to coordination and spatial orientation from the vestibular system to the cerebellum [37]. In other words, if the secondary VCT is affected, it leads to disruptions in the transmission of vestibular information to the cerebellum. Consequently, impaired communication can result in ataxia, because the cerebellum plays a crucial role in coordinating motor activity and maintaining balance [37,43]. In 2016, Barmack reported the anatomy and function of mossy fibers in the vestibulocerebellum [44]. The researcher suggested that secondary vestibular neurons from the vestibular nuclei to the cerebellum influenced movement by integrating visual and neck proprioceptive signals and cerebellar signals [44]. Consequently, secondary vestibular neurons play a role in sensory-motor adaptation, and injury to secondary vestibular neurons from the vestibular nuclei to the cerebellum can induce ataxia [44,45]. The results of these previous studies are consistent with those of the current study, showing a difference in ataxia signs according to the reconstruction rate of secondary VCT in patients with cerebellar hemisphere injury.

Many studies have used the pegboard test to evaluate hand function in relation to fine motor dexterity and coordination [15,16,17,32,46]. They referred to the CST as the major neural tract in the human brain for hand function as assessed using the pegboard test [15,16,17]. Although hand function is not directly related to VCT, it indirectly relies on intact sensory and motor pathways [32,47,48]. The pegboard test is used to evaluate hand function, including hand movement control, motor coordination, and spatial orientation [32,33]. These functions are controlled by the VCT, which contributes to overall motor coordination and spatial orientation, which are fundamental for performing tasks evaluated by the pegboard test [32,47,48]. Therefore, any disruption or dysfunction of the VCT can potentially affect the motor coordination required to perform the pegboard test [32,47,48]. For example, individuals with cerebellar injury affecting the vestibulocerebellar system may show difficulties in fine motor control and precise hand–eye coordination during the test [32,49]. Alternatively, the results of the pegboard test may be decreased by cerebellar symptoms such as ataxia caused by the deterioration of motor coordination, balance, and movement control due to cerebellar injury. Consequently, we suggest that injury to the VCT or cerebellar symptoms following cerebellar stroke affect hand function, as evaluated using the Purdue pegboard test. 

However, this study has several limitations. First, this was a case series involving six patients. Therefore, it is difficult to compare ataxia signs, hand function, and the DTI parameters of VCTs. Therefore, it is necessary to conduct research with a large number and a variety of patients for investigating and generalizing this study in the future. Second, it is difficult to conclude that there is an association between hand function and VCTs because no direct studies have investigated the relationship between hand function and VCT according to cerebellar injury, and this study only evaluated the Purdue pegboard test using a sequence of dominant and non-dominant hand, excluding both hands. Finally, statistical analyses were not performed because of the small sample size. Therefore, it is difficult to conclude and generalize the relationship between VCTs and symptoms caused by cerebellar injury, such as signs of ataxia and hand dysfunction. We suggest that these relationships and differences should be studied using statistical procedures in the future. In particular, it is necessary to evaluate ataxia using an objective assessment tool such as the scale of the assessment and rating of ataxia (SARA), and compare with these quantified clinical functional data and the DTI parameters of VCTs, such as fractional anisotropy (FA), mean diffusivity (MD), and tract volume. 

In conclusion, we report differences in ataxia signs, hand function using the Purdue pegboard test, and other cerebellar symptoms according to the results of calculating the injury rate of VCTs using DTI tractography in patients with quadriparesis following cerebellar stroke. We suggest that ataxia is related to secondary VCTs, and hand dysfunction is also related to VCTs. Therefore, we believe that the current study will be helpful in predicting not only imbalance such as ataxia but also the variety of hand problems according to cerebellar injury. Furthermore, it can provide the data for evaluating vestibular function and applying a clinical intervention strategy from the vestibular point of view for patients with ataxia and hand dysfunction following cerebellar injury.

## Data Availability

The data presented in this study are available on request from the corresponding author. The data are not publicly available due to personal data protection.
